# EC359 Enhances Trametinib Efficacy in *Ras*/*Raf*-Driven Ovarian Cancer by Suppressing LIFR Signaling

**DOI:** 10.3390/biom15101396

**Published:** 2025-09-30

**Authors:** William C. Arnold, Durga Meenakshi Panneerdoss, Baskaran Subramani, Megharani Mahajan, Behnam Ebrahimi, Paulina Ramirez, Bindu Santhamma, Suryavathi Viswanadhapalli, Edward R. Kost, Yidong Chen, Zhao Lai, Hareesh B. Nair, Ratna K. Vadlamudi, Yasmin A. Lyons

**Affiliations:** 1 Department of Obstetrics and Gynecology, University of Texas Health San Antonio, San Antonio, TX 78229, USA; williamcarnold1@gmail.com (W.C.A.); panneerdoss@uthscsa.edu (D.M.P.); subramani@uthscsa.edu (B.S.); mahajanm@uthscsa.edu (M.M.); bebrahimib@rockefeller.edu (B.E.); ramirezp6@uthscsa.edu (P.R.); viswanadhapa@uthscsa.edu (S.V.); kost@uthscsa.edu (E.R.K.); 2 Evestra, Inc., San Antonio, TX 78245, USA; bsanthamma@evestra.com; 3 Mays Cancer Canter, University of Texas Health San Antonio, San Antonio, TX 78229, USA; 4 Greehey Children’s Cancer Research Institute, University of Texas Health San Antonio, San Antonio, TX 78229, USA; cheny8@uthscsa.edu (Y.C.); laiz@uthscsa.edu (Z.L.); 5 Department of Population Health Sciences, University of Texas Health San Antonio, San Antonio, TX 78229, USA; 6 Department of Molecular Medicine, University of Texas Health San Antonio, San Antonio, TX 78229, USA; 7 Department of Molecular and Translational Medicine, Texas Tech University Health Sciences Center, El Paso, TX 79905, USA; hbhaskar@ttuhsc.edu; 8 Audie L. Murphy Memorial Veterans Hospital, 7400 Merton Minter Boulevard, San Antonio, TX 78229, USA

**Keywords:** ovarian cancer, low grade serous ovarian cancer, *Ras*/*Raf* mutations, LIFR, EC359, MAPK, STAT3, trametinib

## Abstract

Ovarian cancer (OCa) remains the most lethal gynecologic malignancy in the United States, with low-grade serous and mucinous subtypes frequently driven by KRAS mutations. These mutations activate downstream MAPK and PI3K/AKT signaling pathways, contributing to tumor progression and resistance to therapy. Although the MEK inhibitor trametinib is used to target these pathways, its efficacy is limited in KRAS-mutant OCa due to compensatory activation of the leukemia inhibitory factor (*LIF*)/*LIF* receptor (*LIFR*) axis. In this study, we evaluated the therapeutic potential of combining trametinib with EC359, a selective *LIFR* inhibitor, in *Ras*/*Raf*-driven OCa models. EC359 significantly reduced cell viability, clonogenic survival, and induced cell death via ferroptosis in vitro. Mechanistic studies revealed that EC359 suppressed trametinib-induced activation of *LIFR* downstream signaling. RNA-seq analysis showed that combination therapy downregulated mitochondrial translation and *MYC* target genes while upregulating apoptosis-related genes. In vivo, EC359 and trametinib co-treatment significantly reduced tumor growth in xenograft and PDX models without inducing toxicity. Our studies identify *LIFR* signaling as a critical vulnerability in *Ras*/*Raf*-mutant and low grade serous OCa. Further, it provides strong preclinical rationale for EC359 and trametinib combination therapy as a new therapeutic strategy for treating *Ras*/*Raf*-driven OCa and low-grade serous OCa.

## 1. Introduction

Ovarian cancer (OCa) remains the most lethal gynecologic malignancy in the United States [1]. Although most patients initially respond to standard therapies, including cytoreductive surgery and platinum-based chemotherapy, nearly 90% experience recurrence and ultimately develop chemotherapy-resistant disease [2]. Epithelial ovarian cancer (EOC) comprises five histopathological and molecular subtypes: high-grade serous (HGSOC, ~70%), endometrioid (ENOC, ~10%), clear cell (CCOC, ~10%), mucinous (MOC, ~3%), and low-grade serous (LGSOC, <5%) [3,4]. MOC and LGSOC are strongly associated with KRAS mutations, which are present in 33–57% of these subtypes and correlate with poor prognosis and chemoresistance [4].

Aberrant activation of the *Ras*/*Raf* signaling cascade including the RAF/MEK/ERK and PI3K/AKT pathways—drives tumor proliferation and survival in *Ras*/*Raf*-mutant ovarian cancers. Targeting the RAF–MEK–ERK pathway with MEK inhibitors, enhances growth inhibition and apoptosis in OCa harboring KRAS or BRAF mutations suggesting their dependence on RAS–RAF–MEK–ERK signaling for survival [5]. In clinical trials MEK inhibitor trametinib (NCT02101788) significantly improved progression-free survival (13.0 vs. 7.2 months) in relapsed LGSOC, supporting its therapeutic potential [6]. Despite this knowledge, effective therapies for *Ras*/*Raf*-driven OCa remain limited, underscoring the need for new targeted approaches.

Leukemia inhibitory factor (*LIF*), a pleiotropic cytokine of the interleukin-6 family, signals through a receptor complex composed of *LIF* receptor (*LIFR*) and *gp130* [7,8]. This axis activates downstream JAK/STAT3, MAPK, AKT, and mTOR pathways, all implicated in OCa progression. Elevated expression of *LIF*, *LIFR* correlates with reduced survival in OCa patients and contributes to chemoresistance and tumor microenvironment (TME) modulation [8,9,10].

Emerging evidence from pancreatic cancer models shows that stromal-derived *LIF* acts in a paracrine manner to promote tumor progression, and that *LIFR* blockade enhances response to chemotherapy [11]. Notably, *LIF* expression is upregulated by oncogene *KRAS*, suggesting a potential link between RAS pathway activation and *LIF/LIFR* signaling [12]. However, the role of the *LIF/LIFR* pathway in *Ras*/*Raf*-mutant OCa, remains poorly defined.

We previously developed EC359, a small-molecule inhibitor that disrupts *LIF*/*LIFR* interaction and inhibits *LIFR*-driven oncogenic signaling [13]. EC359 has demonstrated preclinical efficacy in triple-negative breast, endometrial, renal, and pancreatic cancers [14,15,16,17,18]. In this study, we investigate the therapeutic potential of EC359 in *Ras*/*Raf*-driven OCa. Using multiple preclinical models, we demonstrate that EC359 suppresses *LIFR* signaling, enhances the efficacy of the MEK inhibitor trametinib, and inhibits tumor growth both in vitro and in vivo. Our findings provide a strong rationale for the further development of *LIFR*-targeted therapy in *Ras*/*Raf*-altered OCa.

## 2. Materials and Methods

### 2.1. Cell Culture and Reagents

The OCa cell lines OVCAR8, ES2, OV7, and OV-56 were obtained from the American Type Culture Collection (ATCC, Manassas, VA, USA) and cultured according to ATCC-recommended conditions. All cell lines were routinely tested and confirmed to be free of mycoplasma contamination. Cell line identity was verified by short tandem repeat (STR) DNA profiling. The primary LGSOC cell line OCa-76 was obtained from the Ob/Gyn tissue core under an IRB-approved protocol. OV7 cells harbor *KRAS G12D* mutation; OV56 cells harbor *KRAS G12C* mutation; OVCAR8 cells harbor *KRAS Pro121His* mutation; ES2 harbors a common *BRAF p.V600E* mutation (Source: cellosaurus.org). OCA-76 represents pathologically confirmed LGSOC based on its expression of markers consistent with serous Müllerian origin (*CK7*, *PAX8*, *ER*, *WT1*), absence of aberrant *p53* expression, a moderate proliferative index (Ki-67~20%), and lack of expression of *CK20*, *GATA3*, and *CD10*. The MEK inhibitor trametinib was purchased from MedChemExpress (Monmouth Junction, NJ, USA). EC359 was developed by Evestra Inc., (San Antonio, TX, USA) and the detailed synthetic protocol has been described in the patent WO 2016/154203 A1.

### 2.2. Cell Viability, Colony Formation, and Apoptosis Assays

Cell viability was assessed using the MTT assay in control, and EC359-treated, OCa cell lines, following standard MTT assay protocols. The effects of control and treatment groups on colony formation and apoptosis were evaluated using previously established methods, as described in prior studies [19].

### 2.3. Western Blotting

OCa cells were lysed using RIPA buffer supplemented with protease and phosphatase inhibitors. Protein lysates were quantified and subjected to Western blot analysis using standard protocols. The following primary antibodies were used: Phospho-Akt (S473), Akt, phospho-mTOR (S2448), mTOR, phospho-S6 (S235/236), S6, phospho-STAT3 (Y705), STAT3 (all from Cell Signaling Technology, Beverly, MA, USA), LIF and LIFR (from Santa Cruz Biotechnology, Dallas, TX, USA), and GAPDH and Vinculin (from Sigma-Aldrich, St. Louis, MO, USA).

### 2.4. RNA Sequencing

RNA was extracted from OVCAR8 cells treated with vehicle, EC359 (150 nM), trametinib (100 nM), or the combination of both agents for 7 h, using the RNeasy Mini Kit (Qiagen, Valencia, CA, USA) according to the manufacturer’s instructions. RNA sequencing and downstream analysis were performed by the UT Health San Antonio Sequencing Core using established protocols as described in our earlier publication [19]. The RNA-seq dataset has been deposited in the Gene Expression Omnibus (GEO) under accession number GSE306834.

### 2.5. Cell Line-Derived and Patient-Derived Xenograft Studies

All animal experiments were conducted in accordance with protocols approved by the Institutional Animal Care and Use Committee (IACUC) at UT Health San Antonio. Female SCID mice (6–8 weeks old) were obtained from Charles River Laboratories for both cell line-derived xenograft (CDX) and patient-derived xenograft (PDX) studies. For CDX studies, ES2 cells (1 × 10^5^) were subcutaneously injected into the flanks of mice. For PDX studies, OCa-76 tumor cells (1 × 10^6^) were similarly implanted subcutaneously. Once tumors were established, animals were randomized into four treatment groups: vehicle control, EC359 (5 mg/kg, intraperitoneally, 5 days per week), trametinib (0.3 mg/kg, orally, 3 days per week), or a combination of both agents. EC359 dose 5 mg/kg/ip was selected based on dose ranging and toxicity studies that showed that 5 mg/kg has no toxicity [19]. The selected dose is low end of EC359 MTD dose. Similarly, trametinib dose was selected based on published studies 0.3 mg/kg once daily as a well-tolerated, pharmacologically active dose [20]. Group sizes were determined based on prior experimental results and published studies, using an unpaired model with 80% power and a significance level of *p* = 0.05. Tumor growth was monitored every 3–4 days using caliper measurements, and tumor volume was calculated using the modified ellipsoidal formula: ^1^/_2_ × (length × width^2^). Body weight was also recorded regularly to assess treatment-related toxicity. Toxicity was monitored by weight loss (>20%), signs of acute pain or distress, unable to eat, walk, groom normally, or moribund. We have not observed any of these toxicity signs during study. At the study endpoint, mice were euthanized, and tumors were excised and weighed.

### 2.6. Statistical Ananlyses

Statistical differences between groups were analyzed using unpaired Student’s *t*-tests and one-way ANOVA, as appropriate. All analyses were performed using the GraphPad Prism 10 software. Data are presented as means ± standard error (SE). A *p*-value < 0.05 was considered statistically significant.

## 3. Results

### 3.1. EC359 Treatment Reduces Cell Viability, Clonogenic Survival, and Promote Apoptosis in Ras/Raf-Mutant OCa Cells

**Figure 1:**
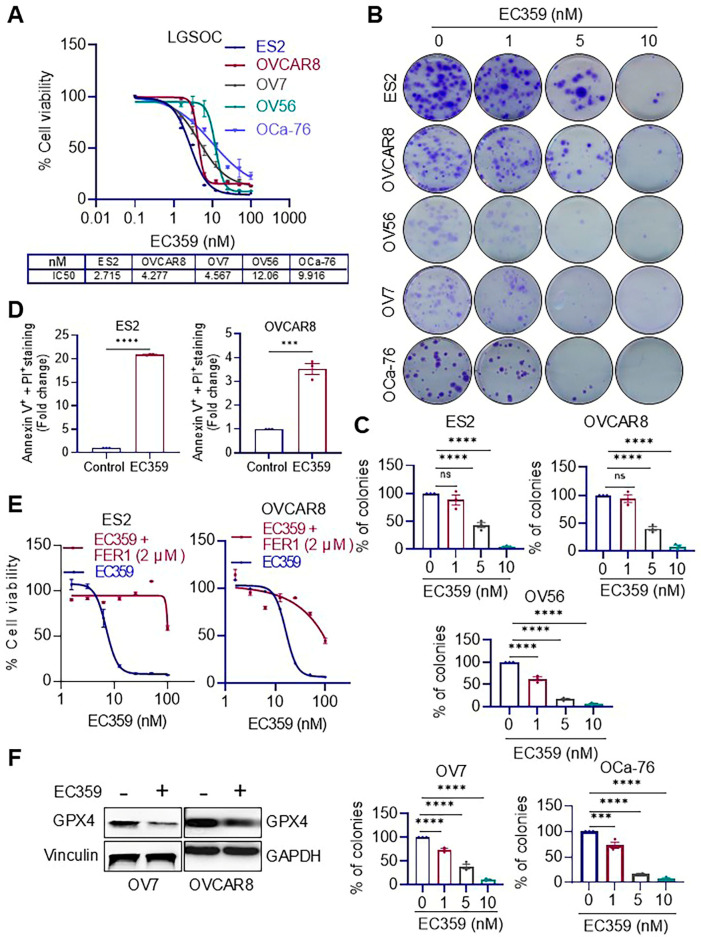
EC359 inhibits cell viability and induces ferroptosis in OCa models. (**A**) MTT assays demonstrate a dose-dependent decrease in cell viability following EC359 treatment across five OCa models (OVCAR8, OV7, OV56, OCa-76, and ES2). (**B**,**C**) EC359 markedly suppresses clonogenic survival in representative cell lines. Representative colony images (**B**) and corresponding quantification (**C**) are shown. (**D**) Annexin V assays reveal a significant increase in apoptotic cell populations upon EC359 (100 nM-48 h) treatment. (**E**) Co-treatment with the ferroptosis inhibitor ferrostatin-1 (Fer-1) rescues EC359-induced loss of cell viability. (**F**) Western blot analysis shows downregulation of the key ferroptosis regulator GPX4 in EC359 (100 nM-7 h) treated cells. Dots represent number of replicates. Data presented as mean ± SEM; significance was determined by Student’ *t*-test; one-way ANOVA; *** *p* < 0.001; **** *p* < 0.0001; ns, not significant.

Among OCa subtypes, *KRAS* mutations are frequently observed in borderline serous tumors, low-grade serous ovarian cancer (LGSOC), and mucinous ovarian cancer (MOC), with reported mutation frequencies ranging around 33–41%, 35–54%, and 57.1%, respectively. KRAS mutations are associated with poor prognosis and chemoresistance in OCa. Recent studies have also demonstrated that *Ras* mutations enhance LIF signaling, suggesting a potential therapeutic vulnerability. To investigate the functional relevance of targeting LIFR signaling in *Ras*/*Raf*-mutant OCa, we evaluated the efficacy of the LIFR inhibitor EC359. The specificity of EC359 to LIFR has been previously demonstrated in our published work using LIFR knockout (KO) cells [13]. We tested EC359 in three Ras-mutant OCa models (OVCAR8, OV7, OV56), one Raf-mutant model (ES2) and one LGSOC primary cell line (OCA-76). MTT assays revealed that EC359 significantly reduced cell viability in all five models, with IC_50_ values ranging from 2 to 12 nM (Figure 1A). EC359 also markedly suppressed clonogenic survival (Figure 1B,C). To determine whether reduced viability was associated with cell death, we performed Annexin V assays. The results showed a significant increase in apoptotic cells following EC359 treatment (Figure 1D). Recent studies have shown that the inhibition of LIF signaling can promote ferroptotic cell death [19]. To investigate whether EC359-induced cell death involves ferroptosis, we co-treated OCa cells with Ferrostatin-1 (Fer-1), a selective ferroptosis inhibitor. Co-treatment with Fer-1 effectively rescued cell viability, indicating that ferroptosis contributes to the cytotoxic effects of EC359 (Figure 1E). Consistent with this, Western blot analysis demonstrated downregulation of key ferroptosis regulator GPX4, in EC359-treated cells (Figure 1F). Collectively, these findings indicate that LIFR signaling is essential for the survival and proliferation of *Ras*/*Raf*-mutant and low grade OCa cells and that EC359 exerts its antitumor effects through induction of both apoptosis and ferroptosis.

### 3.2. EC359 Suppresses LIFR Downstream Signaling and Inhibits Tumor Growth in Ras/Raf-Mutant OCa Xenografts

**Figure 2:**
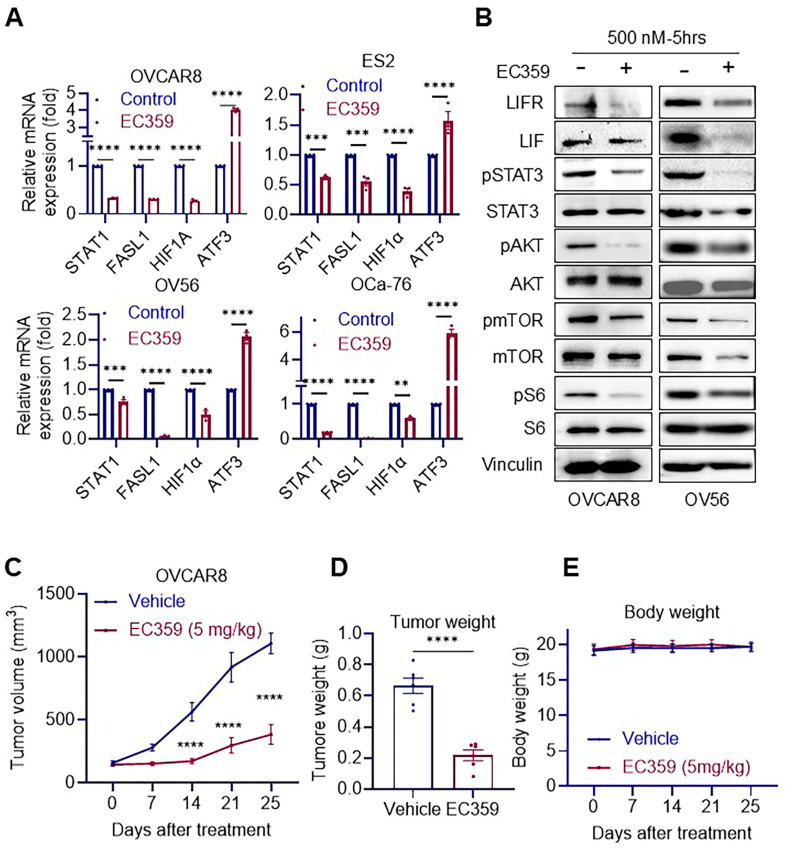
EC359 inhibits LIFR downstream signaling and reduces tumor growth in vivo. (**A**) RT-qPCR analysis showing reduced expression of LIFR downstream target genes in *Ras*/*Raf* mutant cell lines following EC359 (100 nM-7 h) treatment compared to control. (**B**) Western blot analysis demonstrating decreased levels of phosphorylated STAT3 and other LIFR pathway effectors in EC359-treated cell lines. (**C**) Tumor volume measurements over time show significant reduction in tumor growth in EC359-treated mice compared to vehicle-treated controls bearing OVCAR8 xenografts. (**D**) Final tumor weight at the end of the treatment period was significantly lower in the EC359 group compared to vehicle. (**E**) Body weight of mice treated with EC359, or vehicle remained unchanged throughout the study, indicating lack of overt systemic toxicity. Data presented as mean ± SEM; significance was determined by two-way ANOVA; ** *p* < 0.01; *** *p* < 0.001; **** *p* < 0.0001.

To evaluate the impact of LIFR inhibition, *Ras*/*Raf*-mutant OCa cells were treated with EC359 or vehicle control, and analyzed at both the transcriptional and translational levels using RT-qPCR and Western blotting. To assess transcriptional changes, we examined the expression of established LIFR target genes. EC359 treatment significantly downregulated *STAT1*, *FASL1*, and *HIF1α*, while upregulating ATF3, a gene typically repressed by LIFR signaling (Figure 2A). At the protein level, EC359 markedly reduced the phosphorylation of key signaling mediators, including mTOR, STAT3, and S6, confirming effective inhibition of LIFR-mediated signaling pathways (Figure 2B). To investigate the in vivo efficacy of EC359, we established OVCAR8 xenografts in female SCID mice. Mice were randomized to receive either vehicle or EC359 treatment. EC359 significantly suppressed tumor growth and reduced final tumor weights compared to vehicle controls, without affecting overall body weight (Figure 2C–E). Collectively, these findings demonstrate that *Ras*/*Raf*-mutant OCa cells depend on LIF/LIFR autocrine signaling for survival and proliferation. EC359 effectively disrupts this pathway, leading to inhibition of downstream signaling and suppression of tumor growth both in vitro and in vivo.

### 3.3. Trametinib Induces Aberrant Activation of LIFR Signaling in Ras/Raf-Mutant Ovarian Cancer Cells, Which Is Effectively Suppressed by EC359

**Figure 3:**
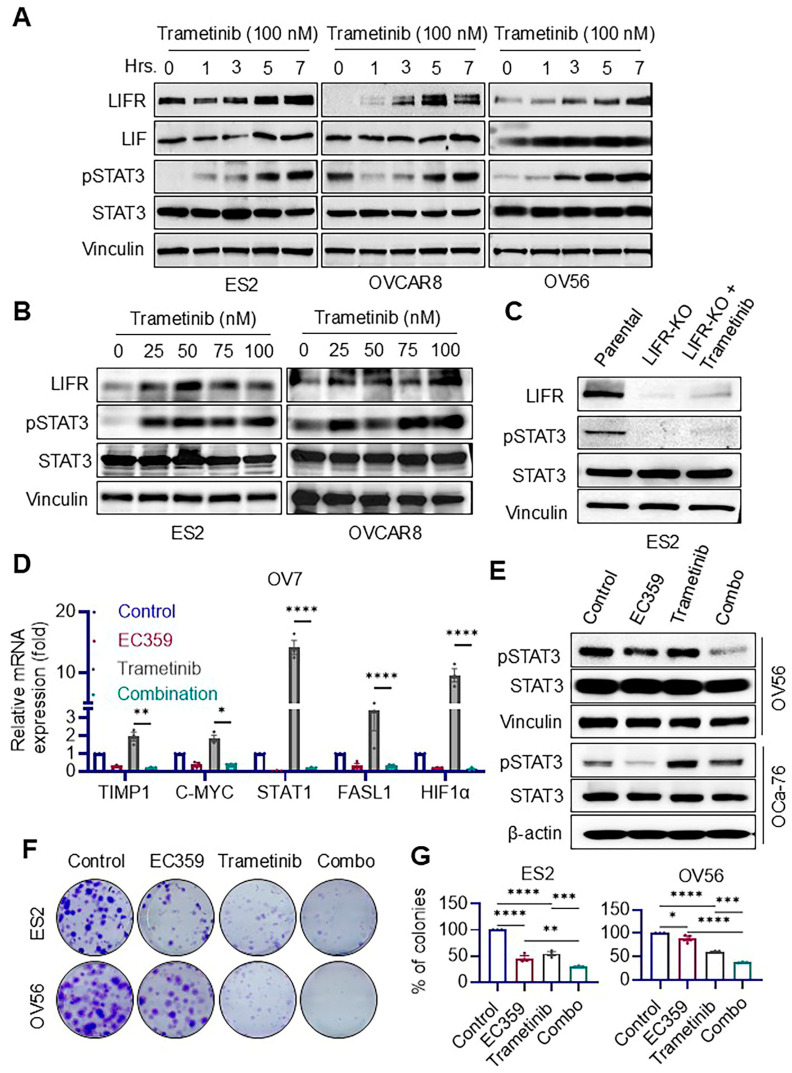
EC359 attenuates trametinib-induced LIFR signaling and enhances therapeutic efficacy in OCa cells. (**A**,**B**) Western blot analysis of multiple OCa cell lines shows a time- (**A**) and dose- (**B**) dependent increase in LIFR and phospho-STAT3 levels following trametinib treatment. (**C**) Western blot analysis of LIFR knockout (LIFR-KO) cells treated with trametinib (100 nM-7 h) reveals no induction of phospho-STAT3 in the absence of LIFR, confirming its requirement for trametinib-induced STAT3 activation. (**D**) RT-qPCR analysis in OV7 cells shows that EC359 suppresses LIFR downstream target gene expression, supporting its inhibitory effect on LIFR signaling. (**E**) Western blot analysis in OV56 and OCa-76 cells demonstrates that EC359 attenuates trametinib-induced upregulation of LIFR and pSTAT3, confirming its role in blocking LIFR-mediated signaling. (**F**,**G**) Colony formation assays reveal that combination treatment with EC359 (ES2-1 nM; OV56-5 nM) and trametinib (ES2-5 nM; OV56-10 nM) significantly reduces colony-forming ability compared to trametinib alone. Representative images (**F**) and quantification of colony formation (**G**) are shown. Data presented as mean ± SEM; significance was determined by two-way ANOVA; * *p* < 0.05; ** *p* < 0.01; *** *p* < 0.001; **** *p* < 0.0001.

Trametinib, a MEK inhibitor, is known to be effective against tumors harboring Ras and Raf mutations by targeting hyperactive MEK signaling. In this study, we investigated its impact on LIF/LIFR signaling in *Ras*/*Raf*-mutant OCa cells. Western blot analyses revealed that trametinib treatment led to a time- and dose-dependent increase in LIFR expression and phosphorylation of STAT3 across multiple OCa cell lines (Figure 3A,B). This trametinib-induced activation of STAT3 was abolished in *LIFR* knockout (*LIFR-KO*) cells, confirming that LIFR is essential for mediating this response (Figure 3C). To determine whether inhibition of *LIFR* could counteract this feedback activation, we examined the effect of EC359, a small molecule LIFR inhibitor. RT-qPCR analysis in OV7 cells demonstrated that EC359 downregulated LIFR downstream target genes, indicating effective transcriptional suppression of LIFR signaling (Figure 3D). Western blot analysis in OV56 and OCa-76 cells further showed that EC359 blocked trametinib-induced upregulation of LIFR and pSTAT3, confirming that it attenuates LIFR-mediated signaling at the protein level (Figure 3E). Functionally, colony formation assays revealed that the combination of EC359 with trametinib significantly reduced the clonogenic survival of *Ras*/*Raf*-mutant OCa cells compared to trametinib alone (Figure 3F,G). The observed synergy between trametinib and EC359 is mechanistically linked to the aberrant activation of LIFR signaling specifically in *Ras*/*Raf*-mutated contexts. To address whether this mechanism extends to wild-type Ras OCa models (SKOV3, OVCAR3), we tested LIFR activation in those cells and found no evidence of LIFR activation (Supplementary Figure S1). These findings suggest that trametinib paradoxically induces LIFR signaling in *Ras*/*Raf*-mutant OCa cells, potentially contributing to adaptive resistance, and that co-treatment with EC359 effectively suppresses this compensatory pathway, enhancing anti-tumor efficacy.

### 3.4. Combination Therapy with Trametinib and EC359 Modulates Transcriptional Programs Associated with Apoptosis and Ribosome Biogenesis

**Figure 4:**
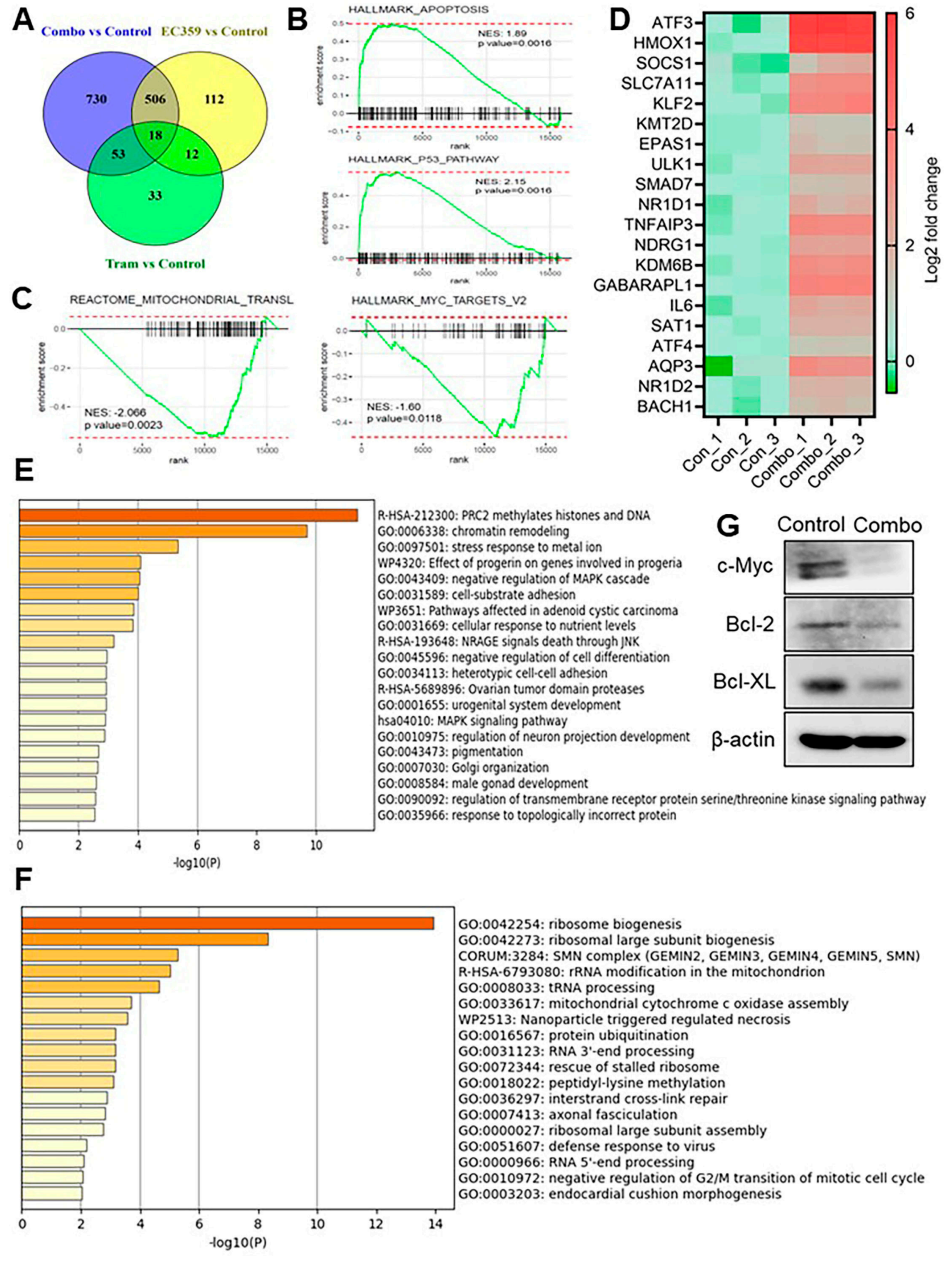
RNA-seq analysis reveals modulation of apoptotic and ribosomal biogenesis pathways in OVCAR8 cells following combination treatment with EC359 and trametinib. (**A**) Venn diagram showing the overlap of differentially expressed genes (DEGs) in OVCAR8 cells treated with EC359, trametinib, or their combination compared to control. (**B**,**C**) Gene Set Enrichment Analysis (GSEA) shows the top significantly enriched pathways in the combination-treated group, including upregulation of apoptotic signaling (**B**) and downregulation of mitochondrial translation and *MYC* target genes (**C**). (**D**) Heatmap show activation of several ferroptosis-related genes following combination treatment (**E**) Bar plot displaying the top enriched pathways (ranked by −log10(*P*)) from genes uniquely upregulated in the combination group, as identified by Metascape. Notable enriched pathways include PRC2-mediated histone and DNA methylation, chromatin remodeling, and stress response to metal ion indicating activation of key tumor-suppressive and regulatory processes. (**F**) Bar plot showing the top enriched pathways (ranked by −log10(*P*)) derived from genes uniquely downregulated in the combination group. These include ribosome biogenesis, ribosomal large subunit biogenesis, rRNA modification, and RNA processing. (**G**) Western blot showing the levels of c-MYC protein levels, and BCL-2 family proteins following combination treatment. Significant genes with fold change > 2 and adjusted *p* value < 0.05 were used for interpreting the biological pathways using GSEA. Fold change values in heatmap were calculated by normalization of FPKM values to those for control.

To better understand the molecular mechanisms underlying the enhanced anti-tumor effects of EC359 and trametinib combination therapy, we performed RNA sequencing on OVCAR8 cells treated with vehicle, EC359, trametinib, or the combination of both agents. Differential gene expression analysis revealed a distinct transcriptional profile in the combination-treated group, with 1307 genes showing ≥2-fold change compared to control. A Venn diagram illustrates the overlap of differentially expressed genes (DEGs) across treatment conditions, with a substantial number of unique DEGs in the combination group (Figure 4A). Gene Set Enrichment Analysis (GSEA) demonstrated that combination therapy significantly upregulated genes involved in apoptotic signaling, while concurrently downregulating genes associated with mitochondrial translation and *MYC* targets, two pathways critical for cellular proliferation and survival (Figure 4B,C). Additionally, RNA-seq analysis confirmed the upregulation of ferroptosis-inducing genes in the combination group when compared to control group indicating a breakdown in ferroptosis defense mechanisms that ultimately contributes to ferroptotic cell death (Figure 4D). To further explore the biological processes uniquely affected by the combination treatment, we performed pathway enrichment analysis using Metascape. Genes uniquely upregulated in the combination group were significantly enriched in pathways related to epigenetic regulation, including PRC2-mediated histone and DNA methylation, chromatin remodeling, and cellular stress response to metal ions, suggesting activation of tumor-suppressive and regulatory processes (Figure 4E). Conversely, genes uniquely downregulated in the combination group were enriched in ribosome biogenesis, ribosomal large subunit assembly, rRNA modification, and RNA 3′-end processing—pathways indicative of suppressed translational activity and protein synthesis (Figure 4F). RNA-seq findings were validated by Western blot analysis, which showed reduced levels of c-Myc and anti-apoptotic proteins following combination treatment (Figure 4G).

Collectively, these findings demonstrate that EC359 and trametinib combination therapy reprograms *Ras*/*Raf*-mutant OCa cells by activating pro-apoptotic and stress response pathways while simultaneously repressing biosynthetic and proliferative programs, contributing to the observed anti-tumor efficacy.

### 3.5. Combination Therapy of EC359 and Trametinib Reduces Tumor Growth in Ras/Raf Mutant OCa Xenografts

**Figure 5:**
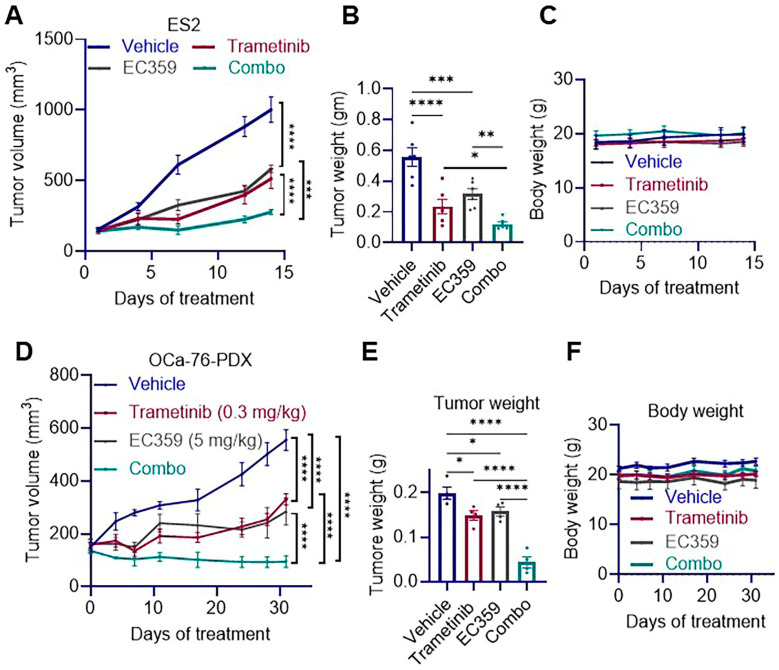
EC359 enhances the antitumor efficacy of trametinib in OCa xenograft and PDX models. (**A**,**B**) In the ES2 xenograft model, combination therapy with EC359 and trametinib led to a significant reduction in tumor volume (**A**) and final tumor weight (**B**) compared to control and single-agent treatments (*n* = 6 tumors). (**C**) Body weight remained stable across all treatment groups, indicating good tolerability. (**D**,**E**) In the OCa-76 PDX model, combination therapy significantly decreased tumor volume (**D**) and tumor weight (**E**) relative to control or monotherapies (*n* = 4 tumors). (**F**) No significant changes in body weight were observed among treatment groups, confirming treatment safety in vivo. Data presented as mean ± SEM; significance was determined by two-way ANOVA; * *p* < 0.05; ** *p* < 0.01; *** *p* < 0.001; **** *p* < 0.0001.

To assess the in vivo efficacy of EC359 and trametinib combination therapy in *Ras*/*Raf*-mutant OCa, we utilized two xenograft models. In the ES2 CDX model harboring Ras mutations, SCID mice were randomized into treatment groups (*n* = 6 tumors/group) and received intraperitoneal EC359, oral trametinib, or their combination. The combination therapy significantly suppressed tumor growth, as evidenced by reduced tumor volume and final tumor weight compared to either single-agent treatment or control (Figure 5A,B). Body weight remained consistent across all treatment groups, indicating good tolerability (Figure 5C). To further validate these findings, we employed the OCa-76 PDX model, which represent LGSOC. Similar to the ES2 model, combination therapy markedly reduced tumor volume and tumor weight compared to monotherapies or control (Figure 5D,E), with no significant changes in body weight observed (Figure 5F). Together, these results demonstrate that co-targeting LIFR and MEK pathways using EC359 and trametinib provides superior anti-tumor efficacy in both xenograft and PDX models, without inducing observable toxicity, supporting its potential as a promising therapeutic strategy.

## 4. Discussion

*Ras* and *Raf* mutations are frequently observed in specific subtypes of OCa, including LGSOC, borderline serous tumors, and MOC [5]. These mutations are associated with poor prognosis and resistance to conventional therapies, largely due to persistent activation of the MAPK signaling pathway [21]. Recent studies in pancreatic cancer have also implicated Ras mutations in the upregulation of LIF signaling, which further promotes tumor cell survival and therapy resistance [11]. Our study identifies LIFR signaling as a critical vulnerability in *Ras*/*Raf*-mutant OCa, and LGSOC and demonstrates the therapeutic potential of targeting this pathway using the LIFR inhibitor EC359, both as a monotherapy and in combination with the MEK inhibitor trametinib.

LGSOC and other *Ras*/*Raf*-driven ovarian cancers pose a significant therapeutic challenge due to their intrinsic resistance to chemotherapy and limited treatment options. Our group previously developed and characterized EC359, a selective small-molecule inhibitor that binds to LIFR and blocks ligand interaction [22]. The specificity and efficacy of EC359 have been validated in several cancer types, including Oca [19], endometrial [14,15], pancreatic [16,17], and renal cancers [18]. In this study, we show that EC359 significantly reduced tumor growth in Ras-mutant OVCAR8 xenografts without affecting body weight, signifies its therapeutic potential and tolerability in vivo.

The LIF/LIFR axis has been implicated in several hallmarks of cancer, including enhanced proliferation, immune evasion, chemoresistance, and poor patient survival. Autocrine or paracrine LIF/LIFR signaling loops have been described in various malignancies [23,24,25]. For instance, LIF acts in a paracrine manner in *Ras*/*Raf*-mutant pancreatic cancer to support tumor progression [11], and to promote stemness and invasion in pancreatic cancer [22]. In our study, we demonstrate that Ras- and Raf-mutant OCa cells express both LIF and LIFR, supporting the presence of an autocrine signaling loop. Pharmacologic inhibition of LIFR with EC359 significantly impaired cell viability and clonogenic survival across multiple Ras- and *Raf*-mutant OCa models.

EC359-induced cytotoxicity was associated with ferroptosis, a regulated form of cell death driven by iron-dependent lipid peroxidation. EC359 treatment increased lipid peroxidation in OCa cells as observed by BODIPY-C11 staining [18]. Further, EC359 treatment is promoted mitochondrial morphological changes characteristic of ferroptosis, as observed by electron microscopy [19]. Mechanistically, EC359 treatment suppressed phosphorylation of key downstream effectors of LIFR signaling, including mTOR, STAT3, and S6, indicating effective blockade of oncogenic signaling. In our studies using *Ras*/*Raf* mutant cancer cells, co-treatment with the ferroptosis inhibitor Ferrostatin-1 rescued EC359-induced cell death, and Western blot analysis confirmed downregulation of the ferroptosis regulator GPX4. These findings are consistent with recent reports that LIFR inhibition promotes ferroptosis [18,19] and suggest that LIFR signaling supports redox homeostasis in *Ras*/*Raf*-mutant OCa cells.

Published studies showed that trametinib resistance occur via multiple mechanisms including epigenetic adaptation, compensatory activation of PI3K/AKT or RTKs, and reactivation of the MAPK pathway [26,27,28]. Interestingly, in our studies we observed that trametinib treatment paradoxically upregulated LIF and LIFR expressions, potentially contributing to adaptive resistance. This aligns with previous studies showing feedback activation of survival pathways following MEK inhibition. Importantly, co-treatment with EC359 abrogated trametinib-induced LIFR activation, resulting in enhanced suppression of clonogenic survival and downstream signaling. Transcriptomic analysis further revealed that the EC359 + trametinib combination downregulated genes associated with the *MYC* and mitochondrial translation pathways. Further, combination therapy upregulated apoptosis-related genes, indicating a shift from a proliferative to a pro-death transcriptional program. These molecular changes were consistent with the observed in vivo efficacy of the combination therapy in both Ras- and Raf-mutant xenograft models, where it significantly reduced tumor volume and weight without inducing toxicity.

Our data suggest a favorable therapeutic window for combination therapy, as the doses of EC359 and trametinib used in this study were well tolerated and showed no signs of systemic toxicity. Our data suggest that the tumor-selective activity of this combination therapy arises from the presence of oncogenic *Ras*/*Raf* mutations in cancer cells, which drive constitutive activation of MEK signaling, and tumor cells with *Ras*/*Raf* mutations exhibit autocrine activation of the LIF/LIFR pathway, further sensitizing them to EC359. In contrast, normal cells lack these oncogenic drivers and therefore are less susceptible to combination therapy. Conducting detailed toxicity studies is beyond the scope of this study, and these will be examined in future studies.

Our research findings have important clinical implications for *Ras*/*Raf*-mutant OCa, which are resistant to standard chemotherapy and associated with poor prognosis. By demonstrating that EC359 potently reduces viability and clonogenic survival and induces both apoptosis and ferroptosis in *Ras*/*Raf*-mutant and low grade OCa cells, this work identifies LIFR signaling as a critical therapeutic vulnerability. Further, the ability of EC359 to overcome trametinib-induced compensatory LIFR activation also adds to its translation potential. Collectively, these results support a EC359/trametinib combination therapy in *Ras*/*Raf*-driven OCa and LGSOC.

## 5. Conclusions

In summary, our findings underscore the therapeutic potential of dual targeting the LIFR and MEK pathways in *Ras*/*Raf*-mutant OCa. EC359 effectively disrupts LIFR-mediated survival signaling and enhances the antitumor efficacy of trametinib by counteracting its compensatory upregulation of the LIF/LIFR axis. This combination strategy presents a promising approach to overcoming resistance mechanisms and improving therapeutic outcomes in patients with *Ras*/*Raf*-driven OCa.

## 6. Patents

Evestra Inc. San Antonio has a patent on EC359.

## Data Availability

All data generated for this study is included within this article. RNA-seq data have been deposited in the GEO database with GEO accession number GSE306834.

## Supplementary Materials

The following supporting information can be downloaded at: https://www.mdpi.com/article/10.3390/biom15101396/s1.

## Abbreviations

The following abbreviations are used in this manuscript:

AKT	Protein Kinase B
ANOVA	Analysis of Variance
ATCC	American Type Culture Collection
ATF3	Activating Transcription Factor 3
BRAF	B-Raf Proto-Oncogene, Serine/Threonine Kinase
EOC	Epithelial Ovarian Cancer
ENOC	Endometrioid Ovarian Cancer
FASL1	Fas Ligand 1
GEO	Gene Expression Omnibus
GPX4	Glutathione Peroxidase 4
HGSOC	High-Grade Serous Ovarian Cancer
HIF1α	Hypoxia-Inducible Factor 1-alpha
IC_50_	Half-Maximal Inhibitory Concentration
IP	Intraperitoneal
JAK	Janus Kinase
KRAS	Kirsten Rat Sarcoma Viral Oncogene Homolog
LGSOC	Low-Grade Serous Ovarian Cancer
LIF	Leukemia Inhibitory Factor
LIFR	Leukemia Inhibitory Factor Receptor
MEK	Mitogen-Activated Protein Kinase Kinase
MOC	Mucinous Ovarian Cancer
mTOR	Mechanistic Target of Rapamycin
MTT	3-(4,5-Dimethylthiazol-2-yl)-2,5-Diphenyltetrazolium Bromide
OCa	Ovarian Cancer
OCCC	Ovarian Clear Cell Carcinoma
PI3K	Phosphoinositide 3-Kinase
RNA-seq	RNA Sequencing
RT-qPCR	Reverse Transcription Quantitative PolymeRase Chain Reaction
SCID	Severe Combined Immunodeficiency
SE	Standard Error
STAT3	Signal Transducer and Activator of Transcription 3
STR	Short Tandem Repeat
TME	Tumor Microenvironment
