# Supplementary material for: EC359 Enhances Trametinib Efficacy in Ras/Raf-Driven Ovarian Cancer by Suppressing LIFR Signaling
Source: Biomolecules. 2025 Sep 30;15(10):1396. doi: 10.3390/biom15101396 (PMC12562836; doi:10.3390/biom15101396)
Supplement: Supplementary file 1 [file biomolecules-15-01396-s001.zip › biomolecules-3833548-supplementary.pdf]

Supplementary figure 1

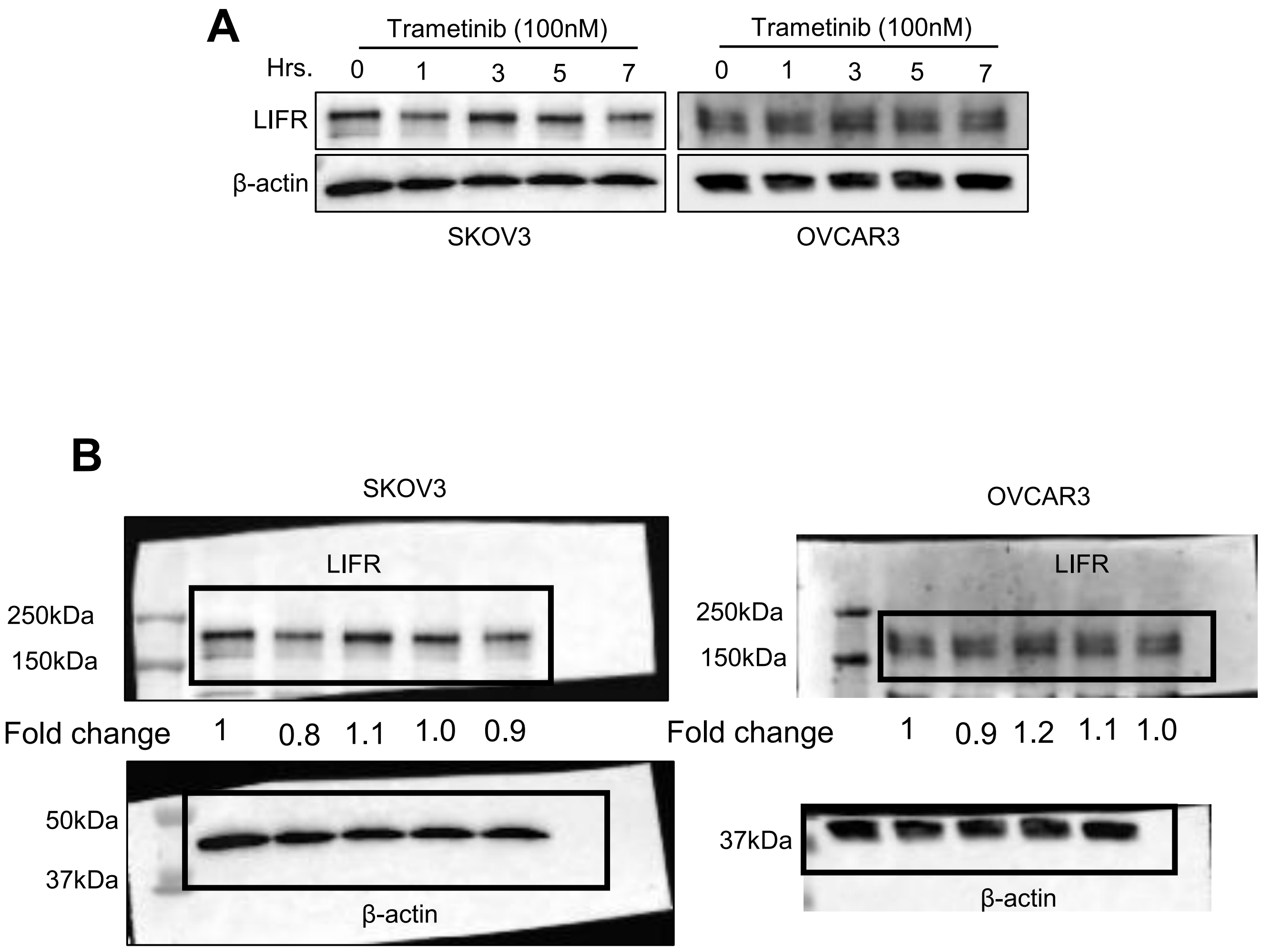

Supplementary figure 1. A, Western blot analysis of SKOV3 and OVCAR3 OCa cell lines shows a time-dependent effect on LIFR levels following trametinib treatment. B, Uncropped Western blots with densitometry were shown.

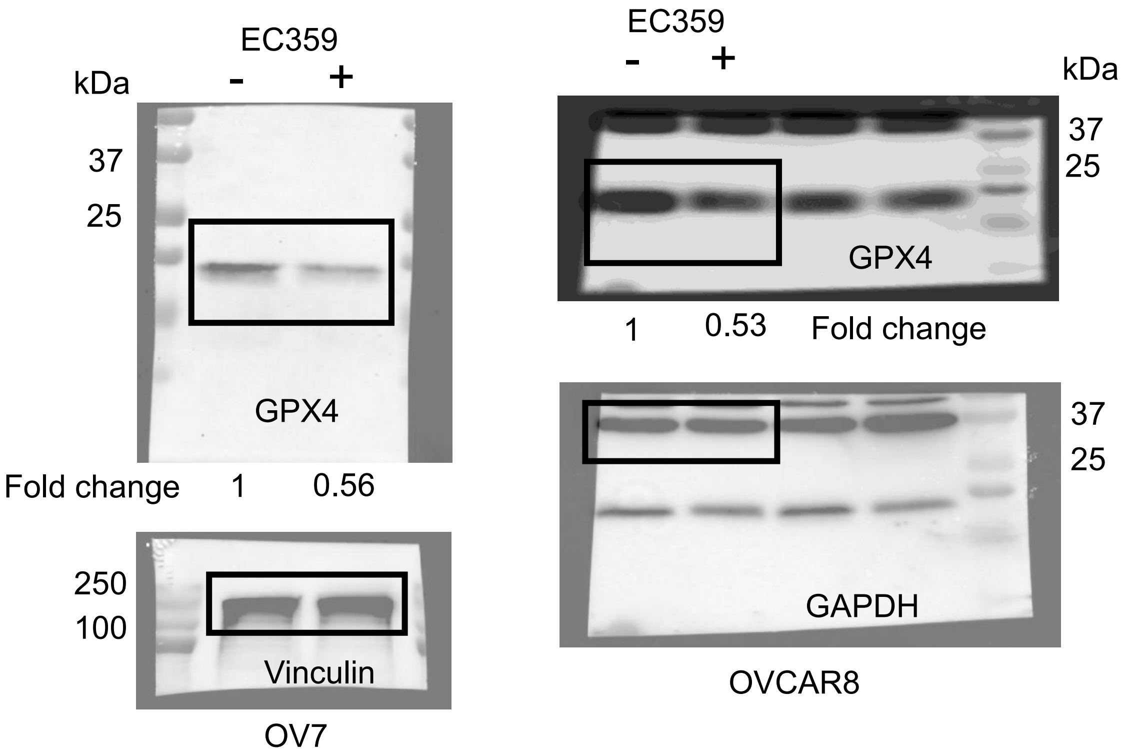

Uncropped Western blots with densitometry for figure 1F were shown.

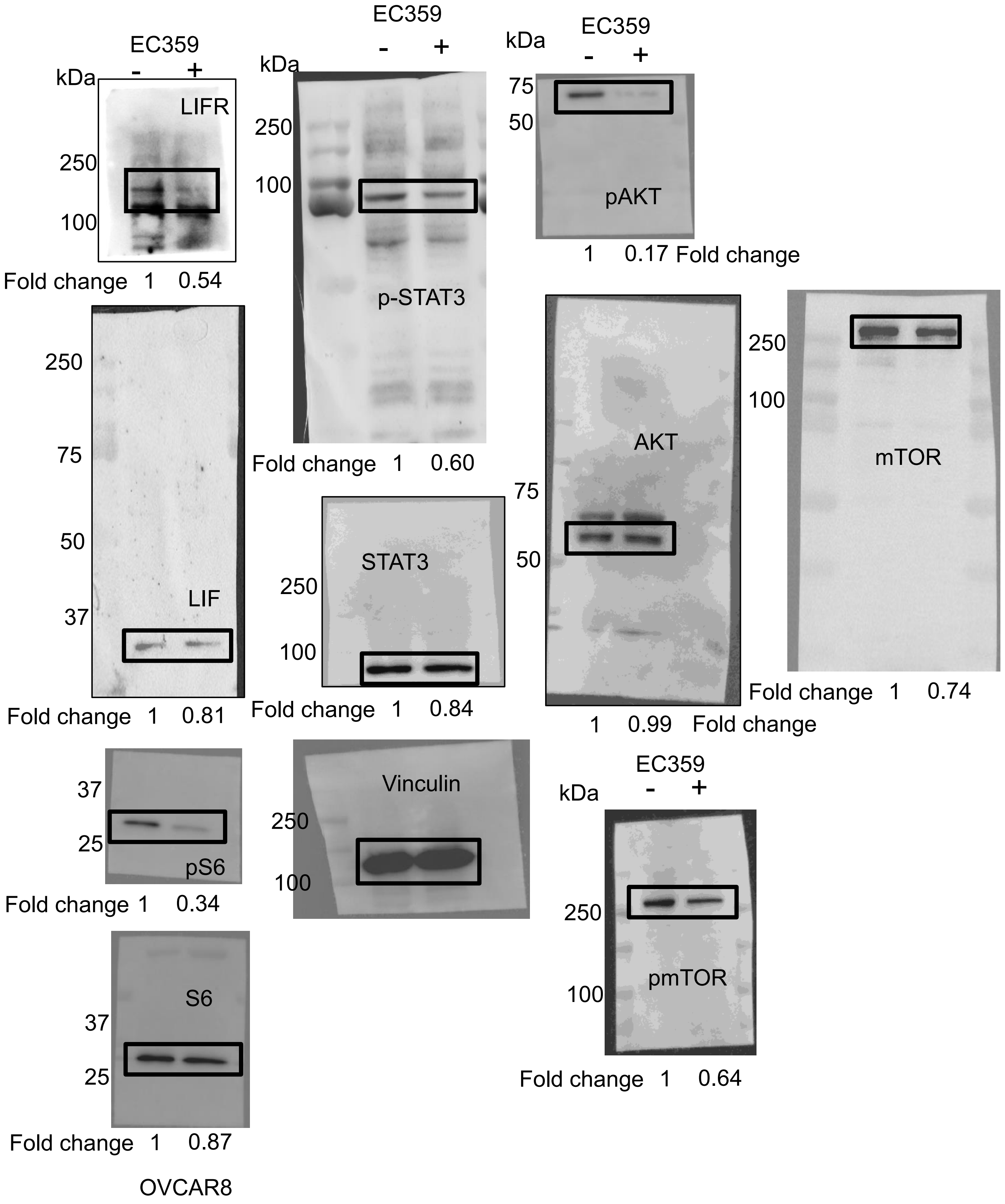

Uncropped Western blots with densitometry of figure 2B were shown.

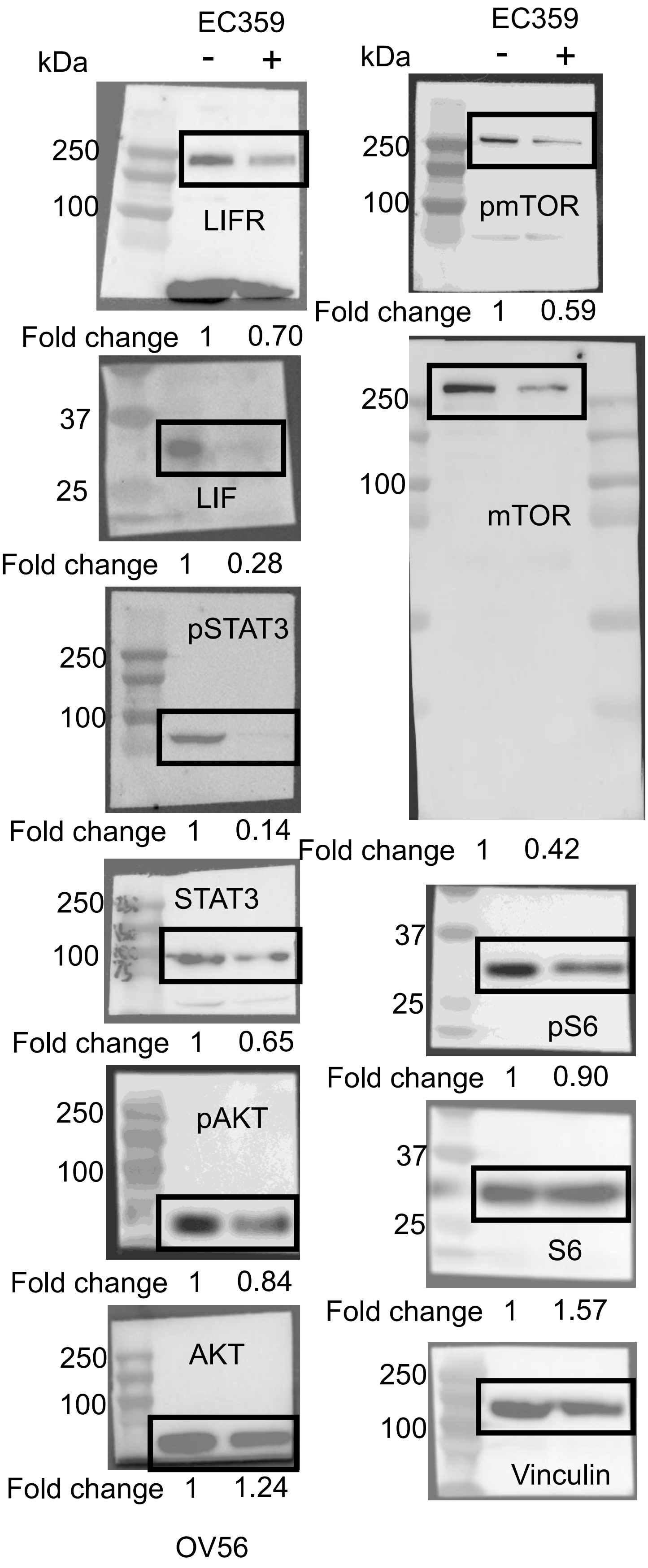

Uncropped Western blots with densitometry for figure 2B were shown.

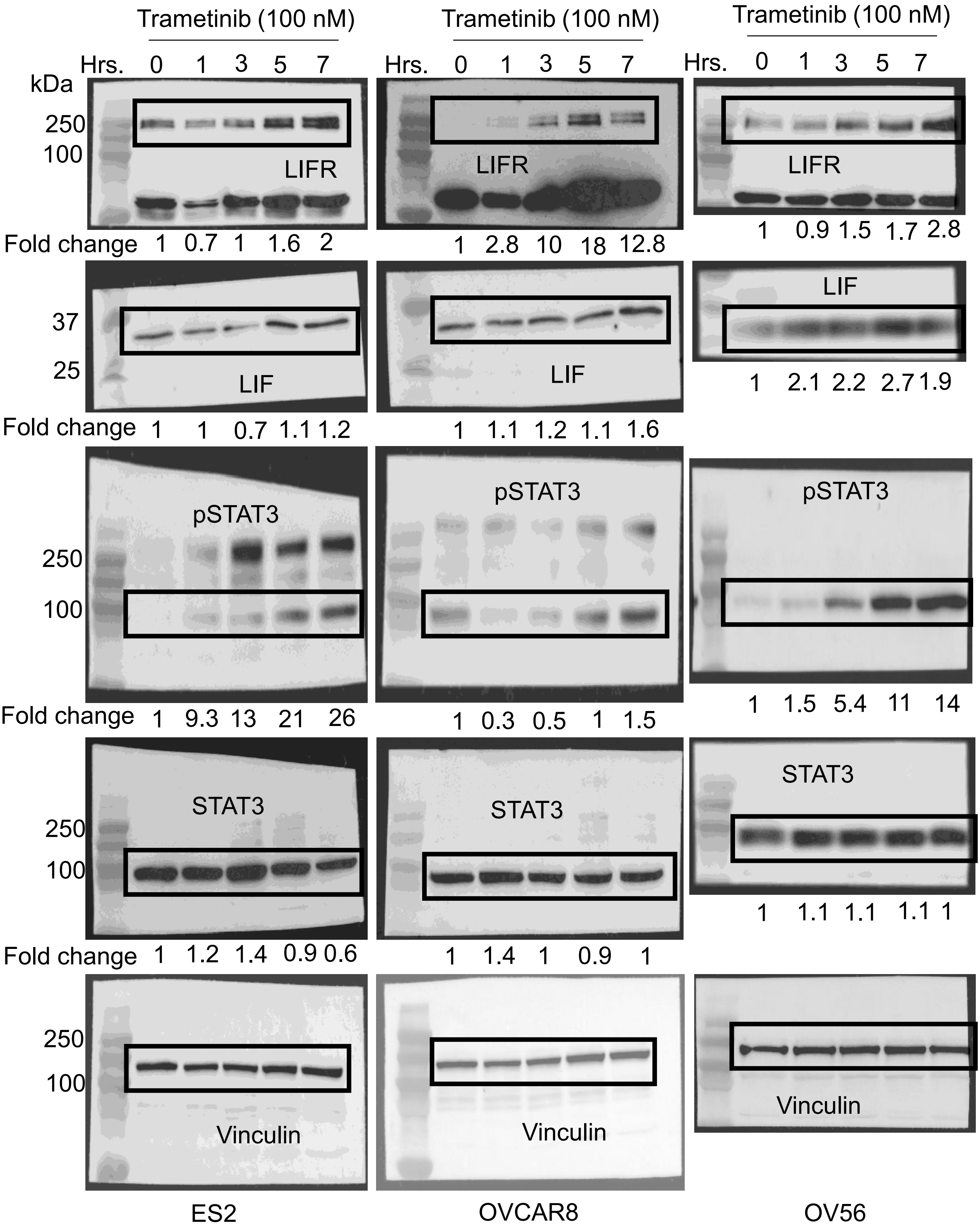

Uncropped Western blots with densitometry for figure 3A were shown.

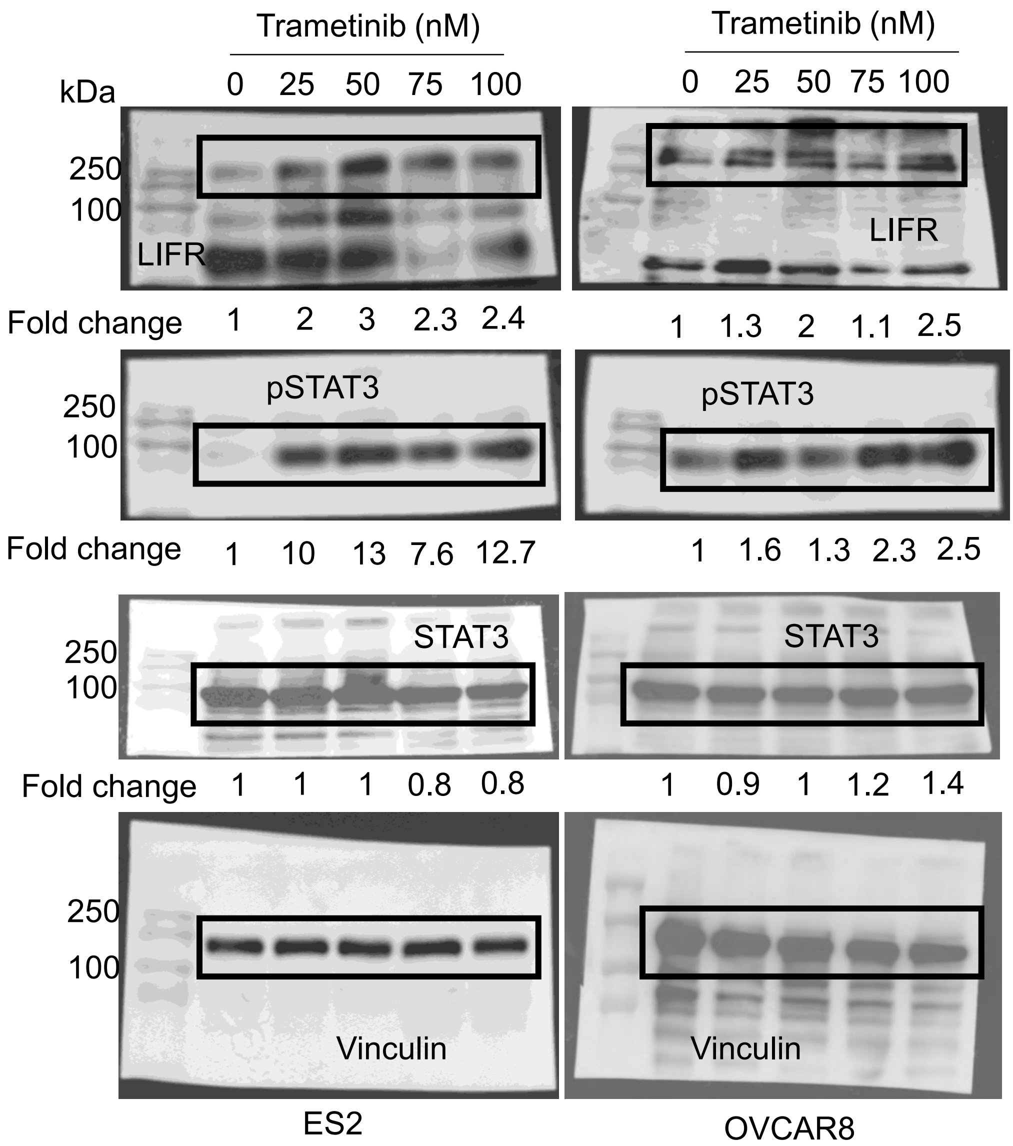

Uncropped Western blots with densitometry for figure 3B were shown.

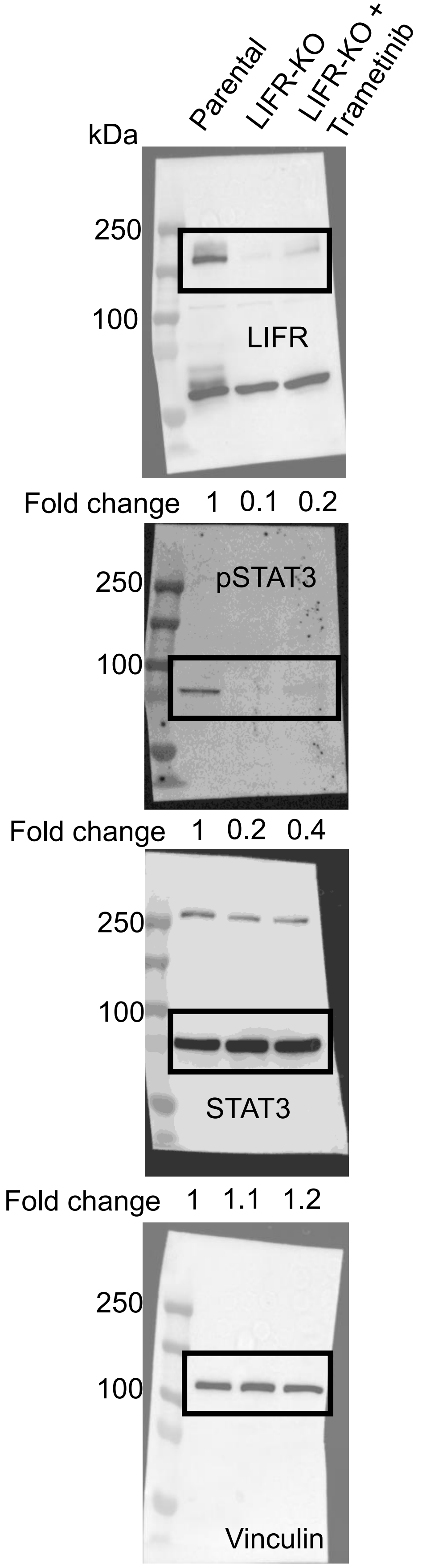

ES2

Uncropped Western blots with densitometry for figure 3C were shown.

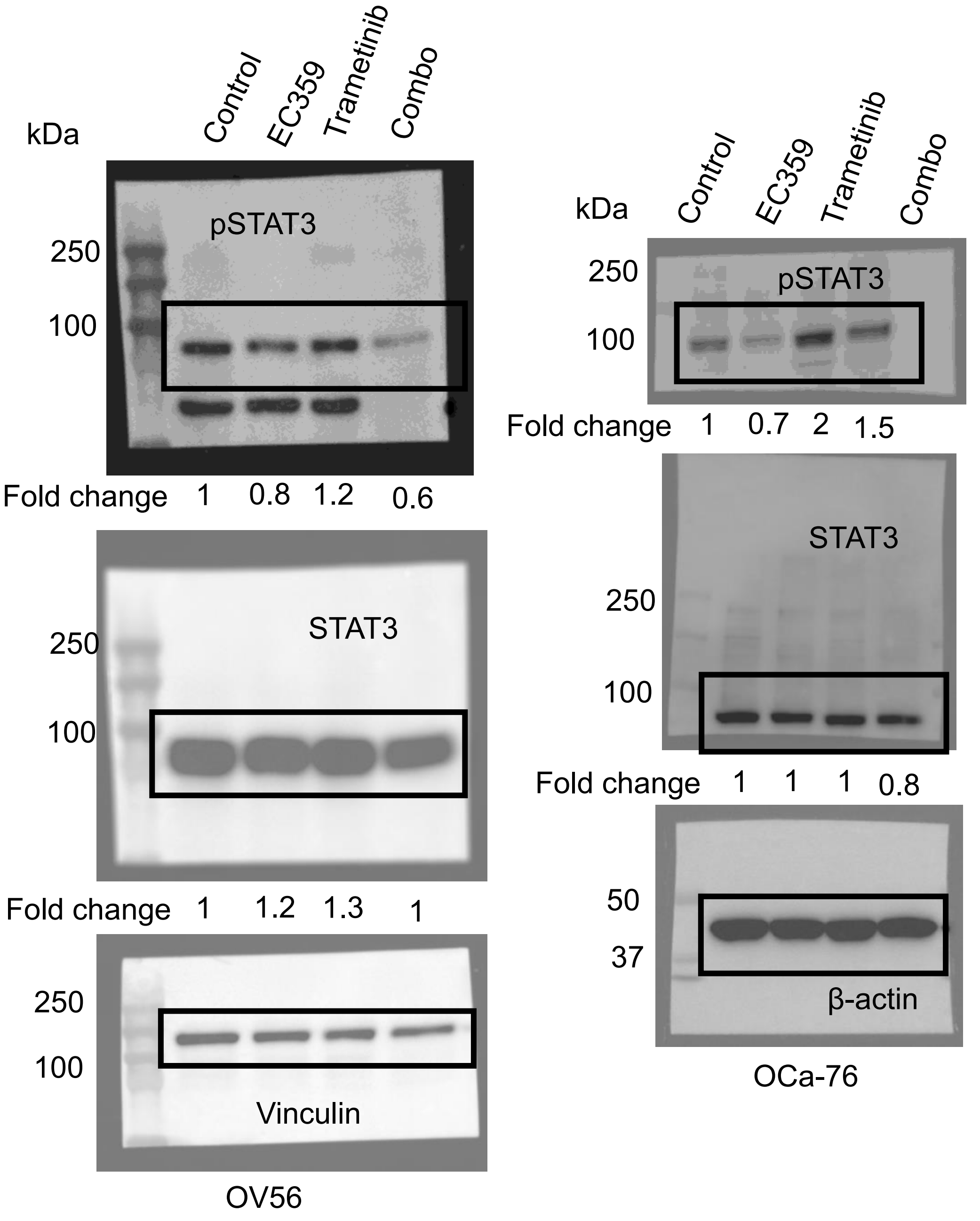

Uncropped Western blots with densitometry for figure 3E were shown.

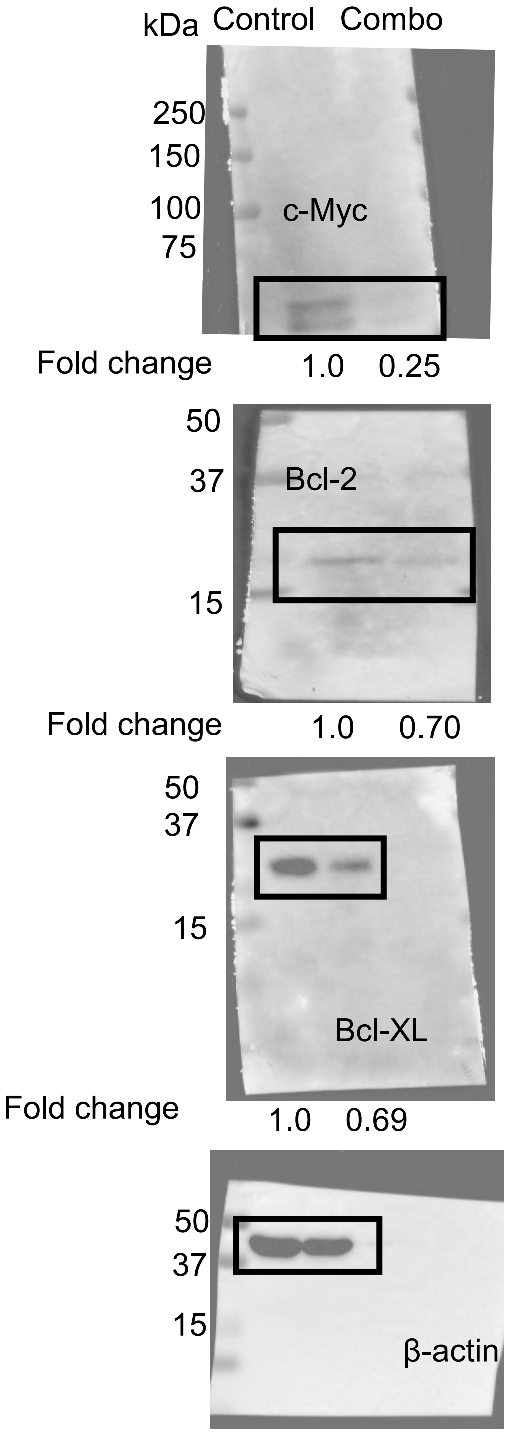

Uncropped Western blots with densitometry for figure 4G were shown.
